# Transdermal optical imaging revealed different spatiotemporal patterns of facial cardiovascular activities

**DOI:** 10.1038/s41598-018-28804-0

**Published:** 2018-07-12

**Authors:** Jiangang Liu, Hong Luo, Paul Pu Zheng, Si Jia Wu, Kang Lee

**Affiliations:** 1 School of Computer and Information Technology, Beijing Jiaotong University, Beijing, 100044 China; 2 The Affiliated Hospital of Hangzhou Normal University, Hangzhou, 310015 China; 3 Dr. Eric Jackman Institute of Child Study, University of Toronto, Toronto, Ontario M5R 2X2 Canada

## Abstract

Human cardiovascular activities are important indicators of a variety of physiological and psychological activities in human neuroscience research. The present proof-of-concept study aimed to reveal the spatiotemporal patterns of cardiovascular activities from the dynamic changes in hemoglobin concentrations in the face. We first recorded the dynamics of facial transdermal blood flow using a digital video camera and the Electrocardiography (ECG) signals using an ECG system simultaneously. Then we decomposed the video imaging data extracted from different sub-regions of a face into independent components using group independent component analysis (group ICA). Finally, the ICA components that included cardiovascular activities were identified by correlating their magnitude spectrum to those obtained from the ECG. We found that cardiovascular activities were associated with five independent components reflecting different spatiotemporal dynamics of facial blood flow changes. The strongest strengths of these ICA components were observed in the bilateral forehead, the left chin, and the left cheek, respectively. Our findings suggest that the cardiovascular activities presented different dynamic properties within different facial sub-regions, respectively. More broadly, the present findings point to the potential of the transdermal optical imaging technology as a new neuroscience methodology to study human physiology and psychology, noninvasively and remotely in a contactless manner.

## Introduction

Human cardiovascular activities are important biological signals. They are convenient and reliable indicators of a variety of physiological and psychological activities and thus are widely used in human neuroscience research^1–3^. For example, during rest, cardiovascular activity is at the baseline level. However, when people are under acute stress, their cardiovascular activity increases, whereas when they are concentrating, their cardiovascular activity decreases. For this reason, researchers have devised various methods to measure cardiovascular activities in human neuroscience research.

One of the methods is to obtain cardiovascular activity signals optically from under the skin. It is well known for almost a century that due to the translucent nature of the human skin, light can penetrate the skin and reemit^4–6^. The reemitted light can be captured externally by optical sensors, from which one can extract blood flow changes beneath the skin. Because the blood flow beneath the skin couples tightly with the cardiovascular change, one can then use the blood flow changes to index cardiovascular activities associated with various physiological and psychological states.

Based on this principle, Photo-plethysmography (PPG) is a relatively inexpensive methodology to non-invasively detect cardiovascular activities^7^. The implementation of PPG is typically dependent of dedicated light sources and the attachment of the sensors to a particular part of the body, primarily the earlobe or the finger, making this imaging methodology still somewhat cumbersome, inconvenient, and invasive.

Recently, researchers have proposed the use of simple video images of the face and the ambient light sources to extract cardiovascular activities information^8–13^. This method is commonly referred to as video plethysmography (VPPG). For example, Poh *et al*.^9,10^ extracted the cardiovascular pulse waves from the webcam-recorded video images of faces by performing the temporal independent component analysis (ICA) on the three color channels (i.e., red, green and blue) of face video records. Further, as an extension of ICA, Tsouri *et al*.^12^ performed a constrained ICA on the three color channels of face video records and avoided the sorting problem of ICA. There are several advantages of this approach over the pre-existing technologies. First, because this approach relies on imaging of the visible light spectrum, one can use the now widely available and inexpensive digital video cameras without the need of any additional equipment. Second, due to the use of the digital video cameras, this approach allows for remote and noninvasive measurements of cardiovascular activities in any part of the exposed human body, mostly the face.

However, one of the problems of video plethysmography is that it confounds ballistic cardiac activities with those of blood flow related cardiovascular activities on the face. Ballistic cardiac activities have been well recognized since the 1930s^14^. It is now well established that cardiac activities can be measured precisely by recording subtle movements of the body (including the face), which is referred to as ballistocardiography or BCG in the literature. The source of BCG is the mechanic movements of the heart due to its rhythmic pumping of the blood. Given this source, the activities of BCG are highly synchronized with cardiac pulses based on the electrocardiography or ECG, as the mechanic pulsations of the heart move the whole body including the face in unison.

In contrast, although the ultimate source of cardiovascular activities on the face is the heart’s pumping actions, their source is a different one. Instead of mechanic movements, facial cardiovascular activities are the result of blood flow in the arterial vasculature and therefore of fluid dynamics in nature. Due to the anatomical structure of the vasculature, the blood travels the arteries that become increasingly narrower and more bifurcated. As a result, the farther the blood is away from the heart, the greater the impendence to the blood flow and the slower the flow speed. Further, due to the elasticity of the arterial walls, original cardiovascular activities are attenuated as they spread though the arterial vasculature. One of the major consequences is the location dependent phase shift of cardiac pulses. A well-known application that capitalizes on this fact is using PPG and ECG signals to estimate blood pressure (pulse transit time or PTT)^15^ that requires the measurement of temporal differences between the peeks of ECG and PPG waves. Unfortunately, in the existing studies using VPPG to measure cardiovascular activities on the face, researchers ignored this crucial difference between the ballistocardiac signals and the cardiovascular signals on the face.

Additionally, in their analyses, they averaged pulsating signals across all the pixels of the entire face^9–12^. However, this averaging approach is problematic because it is based on an assumption that the temporal dynamics of cardiovascular activities in different parts of the face is synchronized. This assumption, however, is not true^3,16,17^. It is now well established that the facial vascular distribution is highly heterogeneous due to the unique network of facial vascular system and differential neural controls by the sympathetic and parasympathetic nervous systems^18,19^. In agreement with this view point, Ghiass *et al*., using another optical imaging method (i.e., infrared imaging), also extracted a vascular network that is heterogeneously distributed across faces^20,21^. As a result, the cardiovascular activity differs in different sub-regions of the face. There are likely temporal differences in the cardiovascular activities in different parts of the face. In addition, signals from some sub-regions may be quite salient, whereas signals from other sub-regions may be fairly faint or even absent.

Revealing such different spatiotemporal patterns may have important advantages. For example, the time course extracted from the sub-regions with the strongest cardiac signal presents a relatively high signal-to-noise ratio (SNR) than those extracted from other sub-regions. More importantly, the region-specific time courses may preserve more subtle and fine temporal features of cardiovascular signals. They therefore can be used to be more accurately measure the dynamic properties of cardiovascular activities.

The spatiotemporal information of facial cardiovascular activity may have important physiological and psychological significances. It is generally accepted that the sympathetic and parasympathetic nervous systems, the two branches of the autonomic nervous system (ANS), are involved in emotion regulation. It has additionally been found that the blood flows of different facial regions are controlled differently by the sympathetic and parasympathetic nervous systems^22^. For example, sympathetic vasodilator neurons predominantly control the blood flow of eyelids, cheeks and chin regions, whereas sympathetic vasoconstrictor neurons mainly control the blood flow in the nose and ears regions. In contrast, the blood flow in the forehead region is regulated by both sympathetic and parasympathetic vasodilators^22^. As the sympathetic and parasympathetic nervous systems play different roles in physiological and psychological regulations with the former activating fight-flight reactions, whereas the latter serving to defuse stress reactions, different physiological or psychological states may have respective distinct ANS signatures^23,24^. In turn, different physiological or psychological states (e.g., fight or flight actions) may engender different spatiotemporal patterns of cardiovascular activities on the face.

For example, a recent study found that unpleasant bitter taste stimulus decreased blood flow in the nose, whereas pleasant taste such as sweet and umami increased blood flow in the eyelids^16^. Another study found that capsaicin significantly increased blood flow in the forehead, eyelids, nose, cheeks, and upper and lower lips, whereas menthol significantly decreased blood flow in the nose but increased that in the eyelids, and upper and lower lips^25^. Thus, if we can capture these spatiotemporal patterns of facial blood flow, we can then use them to decode different physiological or psychological states. In contrast, the VPPG method and the related averaging approach, unfortunately, fail to consider the crucial spatiotemporal cardiovascular information.

**Figure 1:**
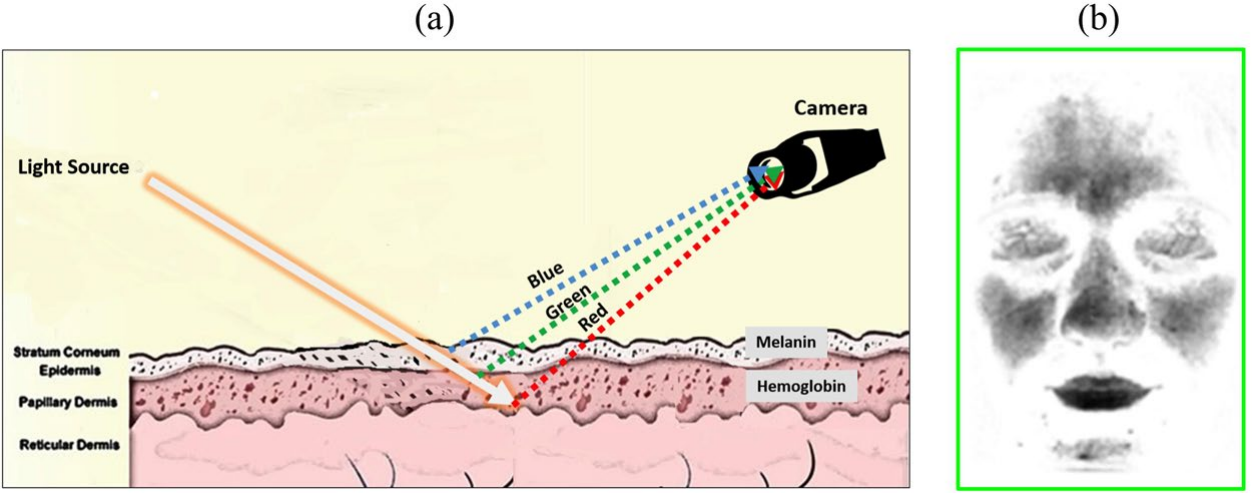
Schematic overview of the acquirement of the transdermal face image. (**a**) The traveling and re-mitting of light through different skin tissues. (**b**) An example of transdermal face image.

The present proof-of-concept study aimed to investigate the spatiotemporal pattern of cardiovascular activities. To this end, we used a novel transdermal optical imaging (TOI) method^26^ that is specifically designed to obtain cardiovascular signals on the face without the interference from the ballistic cardiac activities. This methodology capitalizes on the translucent nature of the skin as mentioned above. As shown in Fig. 1, light re-emits after it travels through different skin tissues, and then can be captured by optical cameras (Fig. 1a). The main chromophores that affect the re-emitted light are melanin and hemoglobin, which show different color signatures, respectively. The TOI methodology uses machine learning to separate the biplanes in the video images that reflect hemoglobin concentrations from those that reflect melanin concentrations, with optimal signal-to-noise ratio (for details, see ref.^26^). As a result, the TOI technology obtains transdermal video images (Fig. 1b) that mainly reflect temporal hemoglobin concentration changes under the epidermis.

To obtain spatiotemporal patterns of such signals, we used independent component analysis (ICA). Different from the studies mentioned above that performed ICA on the signals of three color channels (i.e., red, green and blue) averaged across the whole face^9,10^, the present study performed the temporal ICA on transdermal blood flow dynamics that were extracted from multiple facial sub-regions and mainly included the temporal change information of hemoglobin concentration changes in these regions. Thus, each independent component obtained by ICA should contain both spatial and temporal information that may reflect the underlying facial cardiovascular activity in the sub-regions of the face.

**Figure 2:**
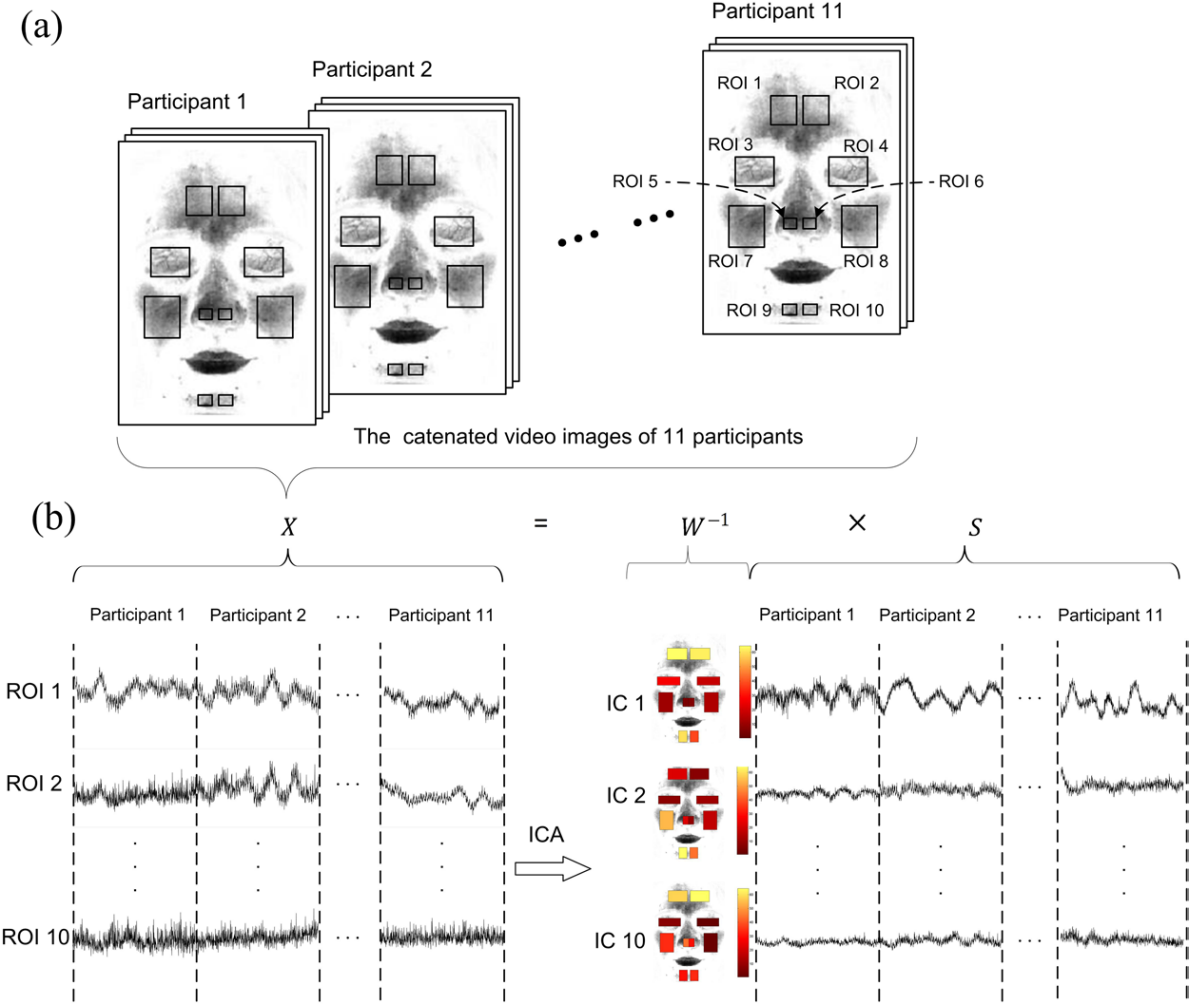
Schematic overview of group ICA. (**a**) The video images of 11 participants are concatenated head-to-tail and the concatenated time courses that include the transdermal facial blood flow are extracted from 10 regions of interests, respectively. (**b**) The group ICA is actually an extended version of Equation 3. Here, each row of the left column shows the concatenated time courses of 11 participants originally extracted from each of the 10 facial ROIs, which is indicated by the corresponding row of Matrix ***X*** in Equation 3. Each independent component decomposed from the original concatenated time courses (i.e., the left column) have two features, namely the temporal dynamics and the spatial distribution. Each row of the right column shows the concatenated temporal dynamics of each independent component across 11 participants, which is indicated by corresponding row of Matrix ***S*** in Equation 3. Each sub-figure of the middle column shows the spatial distribution of each independent component, which is indicated by corresponding column of matrix ***W***^−1^ in Equation 3. It should be noted that, for each independent component, all the time points of its concatenated temporal dynamics have the same spatial distribution. The color map presents the spatial distribution of strength with which the temporal dynamics of one independent component is projected into different ROIs of a face. For an independent component, the more yellow the ROI of its spatial distribution, the stronger strength with which its temporal dynamics is projected into this ROI.

Using Transdermal optical imaging methodology, we divided the face into ten different regions of interests (ROIs) and obtained facial transdermal blood flow data reflecting cardiovascular activities in these ROIs as a function of time (Fig. 2a). We selected these ROIs based on the facial vascular anatomy and the existing evidence about differential neural controls of the facial vasculature^22^. To reduce data dimension and increase the signal to noise ratio, we pooled the image values on each bitplane of each channel to obtain the raw temporal signal for the specific channel in each of the ROIs.

We also concurrently recorded participants’ Electrocardiography (ECG) signals using an ECG system. Then, we used the following spatiotemporal analysis approach to analyze the data. First, we used the group ICA method to decompose temporal signals extracted from all participants’ facial blood flow activities in all facial ROIs into multiple independent components. Thus, such spatiotemporal analysis allows us to explore the spatial distribution pattern of the cardiovascular activity across a face. Second, we selected the independent components whose temporal dynamics contained the information of the facial cardiovascular activities among all participants based on the ECG data. At the same time, we obtained the spatial information provided by these independent components that allowed us to localize the specific facial ROIs where the facial blood flow dynamics optimally indicated the facial cardiovascular activity.

We hypothesized that if the transdermal optical imaging method can indeed capture cardiovascular activities on the face, these activities should correlate with the ECG signals. In specific, specific independent components should be identified to be the best predictors of the ECG signals. Further and more importantly, if cardiovascular activities indeed vary in different parts of the face, our spatiotemporal analysis approach should reveal that these specific independent components show different temporal dynamics in the different sub-regions of the face.

## Results

**Figure 3:**
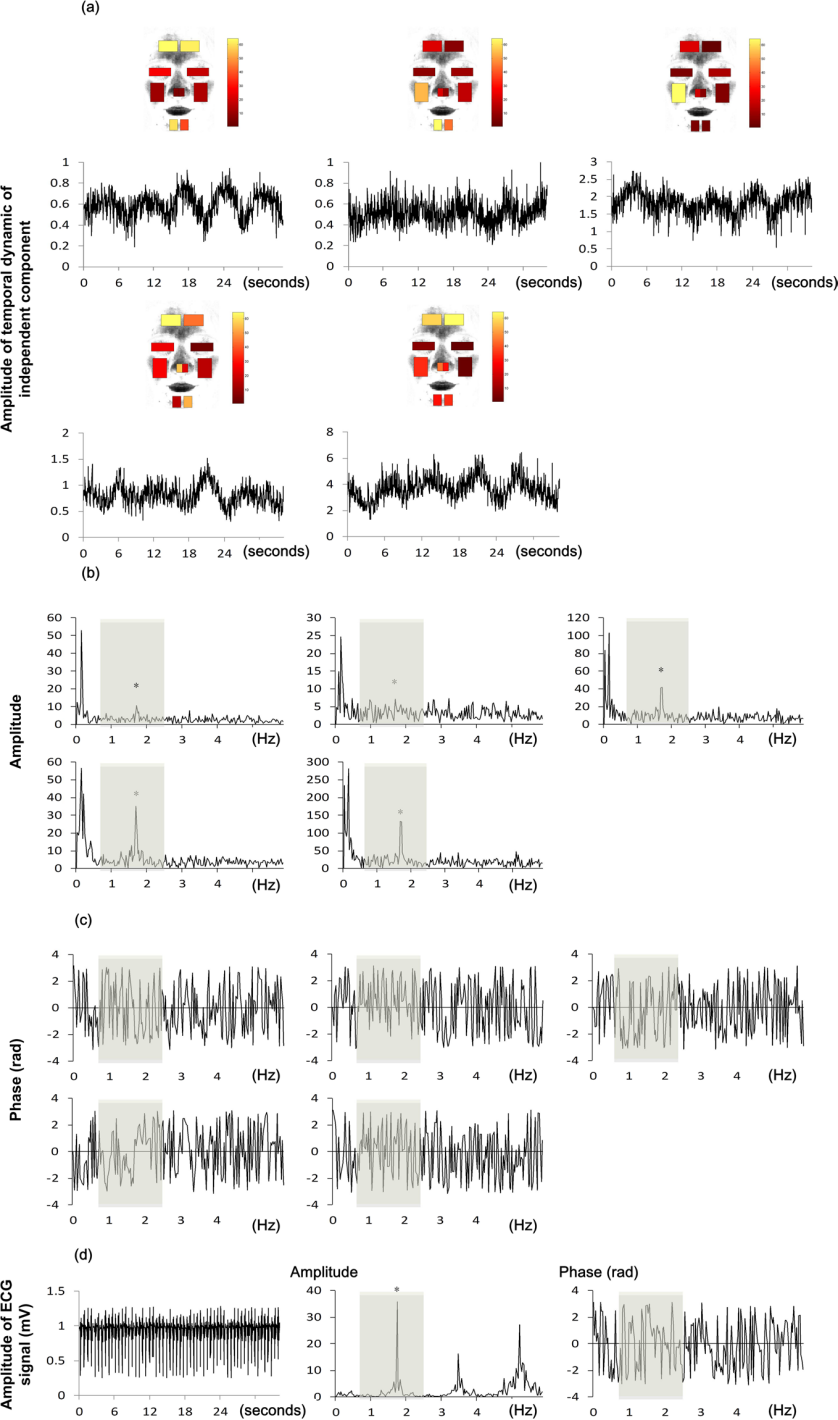
The results of group ICA of an example participant. (**a**) The individual temporal dynamics and spatial distribution of each independent component for this participant. It should be noted that the individual temporal dynamics are segmented from the concatenated temporal dynamics of respective independent components. The color map presents the spatial distribution of strength with which the temporal dynamics of one independent component is projected into different ROIs of a face. For an independent component, the more yellow the ROI of its spatial distribution, the stronger strength with which its temporal dynamics is projected into this ROI. (**b**) The magnitude spectrum of the individual temporal dynamics. (**c**) The phase spectrum of the individual temporal dynamics. (**d**) The time course of ECG signal (left, collected at 200 Hz, resampled to 20 Hz) and its magnitude spectrum (middle), and phase spectrum (right). The shade region indicates the frequency band of 0.7~2.5 Hz.

Figure 3 illustrates the results of data processing for one participant as an example. These results include five independent components (Fig. 3a), their respective magnitude spectrum (Fig. 3b) and phase spectrum (Fig. 3c), ECG signal (Fig. 3d Left), and the magnitude spectrum (Fig. 3d Middle) and phase spectrum (Fig. 3d Right) of the ECG signal.

It should be noted that because the temporal group ICA was used in the present study, the temporal dynamics of each independent component can be segmented into different temporal dynamics for different participants, respectively. Thus, for each independent component, its spatial distribution was invariant for all its segmented temporal dynamics (i.e., for all participants). Additionally, the number of independent components obtained by ICA should be theoretically equal to the number of measured signals (i.e., 10 ICs for 10 ROIs). However, the Fast ICA in the present study did not converge after 5 independent components were decomposed. As a result, only 5 independent components were analyzed here (Fig. 3a).

According to Equation 3, each independent component includes the temporal dynamics and the spatial distribution with the latter reflecting the relative projection strengths of the former at different facial ROIs, respectively. Thus, for each independent component, we obtained the absolute value of its spatial distribution in order to focus on the comparison among these projection strengths. Additionally, for convenience of display, each spatial distribution was also normalized by dividing the maximum absolute value of its 10 projection strengths. Figure 3a shows the temporal dynamics as well as the normalized absolute value of the spatial distribution for each of 5 independent components.

**Figure 4:**
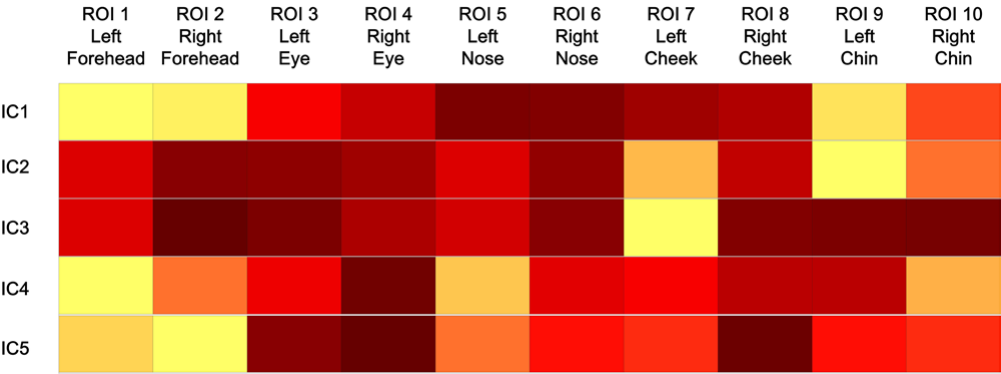
The linear-arranged spatial distributions of five independent (Normalized within independent component). The color map presents the spatial distribution of strength with which the temporal dynamics of one independent component is projected into different ROIs of a face. For an independent component, the more yellow the ROI of its spatial distribution, the stronger strength with which its temporal dynamics is projected into this ROI.

In Fig. 3a, the brighter (i.e., more yellow) ROI indicates that the temporal dynamics of one independent component is projected into this region with stronger strength. As shown by Fig. 3a, the first independent component (IC1) elicited the strongest transdermal blood flow change on the bilateral forehead; the second independent component (IC2) elicited strongest transdermal blood flow change on the left chin; the third independent component (IC3) elicited the strongest transdermal blood flow change on the left cheek; the fourth independent component (IC4) elicited the strongest transdermal blood flow change on the left forehead; and the fifth independent component (IC5) elicited the strongest transdermal blood flow change on the bilateral forehead. For convenience of comparison among these spatial distributions, we transformed them into a 2-D matrix (distribution matrix, Fig. 4). The rows of this matrix indicate the linear-arranged spatial distribution of 5 independent components, respectively, whereas the columns of this matrix present the 10 facial ROIs, respectively.

As shown by this distribution matrix, the signals of some facial regions were composed of more than one independent component. For example, the left forehead presented the strongest projection strengths of the temporal dynamics of IC1 and IC4, and a relatively strong one of IC5; the right forehead presented the strongest projection strengths of the temporal dynamics of IC5, and a relatively strong one of IC1; the left cheek presented the strongest projection strength of the temporal dynamics of IC3 and a relatively strong one of IC2; the left chin presented the strongest projection strength of the temporal dynamics of IC2, and a relatively strong one of IC1. These findings suggested that although the spatial distributions of the independent components were different from each other, some of them carry similar information.

We found significant correlations of magnitude spectrum (band of 0.7~2.5 Hz) between the temporal dynamics of each independent component and the time course of ECG (Table 1 Left columns). These results indicated that all five independent components that were decomposed from the transdermal facial blood flow data indeed included valid information about participants’ cardiovascular activities.

**Table 1:** Correlations of magnitude spectrum (left) and phase spectrum (right) between the temporal dynamics of each independent component and the time course of ECG.

Independent components	Correlations to magnitude spectrum		Correlations to phase spectrum	
	*r*	Statistical value	*r*	Statistical value
IC1	0.273	*t*(10) = 4.679, *p* < 0.001	0.033	*t*(10) = 1.441, *p* = 0.180
IC2	0.378	*t*(10) = 5.636, *p* < 0.001	−0.009	*t*(10) = −0.251, *p* = 0.807
IC3	0.330	*t*(10) = 6.890, *p* < 0.001	−0.013	*t*(10) = −0.302, *p* = 0.769
IC4	0.275	*t*(10) = 3.739, *p* = 0.004	0.017	*t*(10) = 0.476, *p* = 0.644
IC5	0.375	*t*(10) = 7.560, *p* < 0.001	−0.015	*t*(10) = −0.407, *p* = 0.693

We further performed the same correlation analyses between the phase spectrum of the temporal dynamics of the 5 independent components and that of ECG signal, respectively. None of the correlations were significant (Table 1 Right columns). These results suggested that the phases of the cardiovascular activities in the five independent components were asynchronous with the phases of the ECG signals, although the temporal dynamics of all independent components still contained information about cardiovascular activities.

## Discussion

The present study used a conventional digital camera to reliably reveal cardiovascular activities on the face, noninvasively and remotely. We did so in a contactless manner whereby we did not need to attach any sensor to the face. More importantly, we revealed that the cardiovascular activities presented different temporal properties within different facial sub-regions, respectively. To our best acknowledge, the present study is the first to reveal the spatiotemporal features of facial cardiovascular activities based on the transdermal blood flow dynamics.

In the present study, we found that the facial cardiovascular signals could be decomposed into five independent components because their temporal dynamics were independent of each other. We also found that their spatial distributions (i.e., the distribution of the project strengths of the temporal dynamics of each component in all facial regions) also varied greatly from one component to another. More specifically, we found that all temporal dynamics of these five independent components presented cardiac-pulse-like magnitude spectrum band, suggesting that all of them contained information about the cardiovascular activities.

However, at the same time, the phase spectra of these temporal dynamics in this frequency band were completely different from that of ECG signal recorded simultaneously. Such difference in phase spectrum resulted in the asynchrony of the temporal dynamics of the five ICA components. These findings suggested that although the transdermal blood flows in different regions of the face were driven by the same original source, namely the cardiac pulse, the facial cardiovascular activities have different spatiotemporal patterns from that of their original source. These findings suggest that the traditional method of averaging all signals from the entire face is problematic as it obscures the crucial differential spatiotemporal distributions of the cardiovascular activities on the face. The phase shift findings also suggest that the cardiovascular signals obtained through the transdermal optical imaging are not ballistocardiac in nature. Otherwise, no phase shift between the ECG and transdermal signals on the face should be found.

These different patterns of facial blood flow spatiotemporal dynamics might be a result of a number of factors. One potential factor is related to the facial blood vessel distribution. For example, it is well known that blood vessels are long and narrow structures. The arteries supplying the forehead region (superficial temporal artery) and the chin region (maxillary artery) have greatly different terminal branches^27^. Such different blood vessel structures linking the chin and the forehead may exert different influence on the transmission of cardiovascular signals in the blood vessels (e.g., different time delays in different frequencies). As a result, different hemodynamic responses in these two facial regions are observed.

However, the difference in blood vessel distribution does not fully account for the asynchrony among the temporal dynamics of different independent components. In the present study, we found that more than one independent component presented the strongest projection strengths of their temporal dynamics in one region (e.g., the bilateral forehead). In other words, in the same region (without any difference in distribution of blood vessels), there co-exist at least two temporal dynamics that belong to different independent components (and therefore are independent of each other). Thus, the asynchrony among the temporal dynamics of difference independent components may be caused by additional factors.

One such potential factor is the localized neural control. It has been suggested that blood flow in different facial areas is regulated by different vasomotor control mechanisms in the ANS^22^. In the present study, the participants were in resting state when the facial transdermal blood flow signal was recorded. However, many resting state studies based on functional neuroimaging of the cortical blood flow have found that there are spontaneous hemodynamic fluctuations in our brains during the resting state when the brain is presumably not engaged in any specific tasks^28–30^. Especially, it is well established that the brain regions of the default mode network (DMN) even show enhanced hemodynamic activity during the resting state relative to the task-related state^31^. These resting state networks have been suggested to be the baseline state of our brain and are closely related to the processing of sensory-perceptual, emotional, and cognitive information^30^. We therefore speculate that the transdermal blood flows in different parts of the face may also be regulated by the spontaneous fluctuations of the cortical and subcortical structures. If this is the case, our findings suggest that there may exist a close link between the brain hemodynamic activity and the facial blood flow activity. This intriguing hypothesis needs to be tested with specifically designed studies that combine our facial transdermal optical imaging methodology with the conventional functional neuroimaging methodologies such as functional magnetic resonance imaging (fMRI) and functional near-infrared spectroscopy (fNIRS).

It should be noted that because the present study serves as a proof-of-concept of the use of the transdermal optical imaging methodology to analyze facial cardiovascular activities, we only recruited a small number of participants. Future studies need to be conducted that recruit not only a larger sample of participants but also of different skin colors and use different digital video cameras in different lighting conditions to test the robustness of the technology’s application.

With further testing and improvements, the transdermal optical imaging may become a standard tool that capitalizes on the differences in transdermal facial blood flow in different regions of the face to study not only physiological but also psychological activities (e.g., emotional states, stress, and cognitive load). For example, it is well known that different emotional states can be revealed by different dynamic properties of cardiac activities^32,33^, especially for the emotions that vary along the levels of emotion valence and arousal. For example, larger heart rate decelerations occur following unpleasant stimuli presentation as compared to pleasant ones^34,35^. Thus, if the spatiotemporal pattern of cardiovascular activities measured from facial transdermal blood flow varies as a function of emotional states, then we can use transdermal optical imaging as a new method to study emotional regulations in natural settings. The advantage of this method is that we can do so non-invasively, remotely, inexpensively, and sometimes even covertly.

## Conclusion

In conclusion, the present study used a convention digital video camera and a novel transdermal optical imaging technology to extract blood flow data in different parts of the face. We found that facial cardiovascular activities were associated with five independent components reflecting different spatiotemporal dynamics of facial blood flow changes. Our findings suggest that the differential spatiotemporal dynamics may be used to study human physiological and psychological activities in humans. More broadly, the present findings also point to the potential of the transdermal optical imaging technology as a new neuroscience methodology to study human physiology and psychology, noninvasively, remotely and inexpensively.

## Material and Methods

### Participants

Eleven healthy subjects participated in the present study (5 males, 6 females, age 30.05 ± 9.43). None of them took medication that might influence facial blood flow. The present study was approved by the Research Ethics Committee of University of Toronto and participants gave written informed consent prior to participating in the study, and all methods were performed in accordance with the relevant guidelines and regulations.

### Experimental setup

In the present study, we used a Pike F-421 camera (Allied Vision Technologies) to capture transdermal images. The camera was placed about 50 cm from participant’s face and video images were captured at 20 frames per second with the resolution of 910 × 800. Two LED lights were used as the source of illumination. Lights were tested at a National Voluntary Laboratory Accreditation Program (NVLAP) accredited laboratory and the luminous flux difference between them was smaller than 0.15%.

### Experimental procedure and data acquirement

The experiment was carried out in a dark room. Participants were tested individually. During the experiment, participants were instructed to close their eyes, sit still and think of nothing specific. Their face images were collected for 2 minutes. Simultaneously, an electrocardiogram (MP150 analogue/Digital data acquisition system plus ECG100C amplifier, BIOPAC Systems Inc.) was used to record ECG signal at 200 Hz.

### Data preprocessing

Data preprocessing included two steps, namely the acquisition of transdermal images and intensity normalization, both of which were performed using custom software written in MATLAB (The MathWorks, Inc).

#### Acquisition of transdermal facial blood flow images

As mentioned above, we first obtained full color video images of participants’ faces. For each participant, we selected the first 1024 images (approximately 51 seconds) and conducted the analyses as follows. We defined 10 sub-regions (forehead, eyelids, nose, cheeks and chin on both sides of the face) as the regions of interests (ROIs) in the transdermal face images (Fig. 2a). Then, we used the transdermal optical imaging technology^26^ to extract a transdermal image from each frame of the video in each facial ROI. The transdermal image mainly contained information about hemoglobin concentration under the facial epidermis at a particular point in time in each pixel. From this transdermal image, we then used the greyscale intensity to index hemoglobin concentration (i.e., the greyer, the greater hemoglobin concentration) in each pixel of each ROI. We then averaged the image intensities of all pixels within this ROI to derive the hemoglobin concentration intensity data for the ROI in that transdermal image. Finally, by linking the data from each participant’s transdermal images along the temporal dimension, we obtained the mean temporal changes of the transdermal facial hemoglobin concentration changes in each ROI of each participant.

#### Time course extraction and intensity normalization

We then normalized the mean time course of this ROI using the grand mean of pixel intensity across all ROIs of each participant (Equation 1). The grand mean was obtained by averaging the intensities across all pixels of the ten ROIs and all time points of these pixels.

1 $$\begin{array}{c}x^{\prime} (i)=\frac{x(i)}{\mu }\\ \mu =\frac{1}{N}\sum _{i=1}^{10}\sum _{j=1}^{{N}_{i}}\sum _{k=1}^{1024}{t}_{ijk}\\ N=1024\,\sum _{i=1}^{10}{N}_{i}\end{array}$$

where *x*(*i*) indicates the mean time course of *i*th ROI, and *N*_*i*_ indicates the number of pixels in *i*th ROI. ${t}_{ijk}$ represents the intensity of the *k*th time point of the *j*th pixel within the *i*th ROI for a particular participant. The aim of intensity normalization was to remove the inter-individual difference in image intensity, which may result from differences in skin color or tone or other optical properties.

All of the 10 normalized mean time courses from the 10 ROIs were referred to as the original time courses, which would be used in the following spatiotemporal analyses.

### Spatiotemporal analysis of facial cardiovascular activities

#### Temporal independent component analysis

ICA is a computational blind source separation technique for separating multivariate signals into several statistically independent components. Each independent component obtained by ICA method has two features characterizing its spatial distribution and temporal dynamics, respectively. Further, for each independent component, the points of its temporal dynamics have the same spatial distribution pattern, and at the same time each sub-region of its spatial distribution has the same temporal dynamics pattern. Due to such spatiotemporal-invariant property, ICA algorithm is particularly suitable for spatiotemporal analysis.

The objective of ICA is to recover the independent component signal, ***S***, from their linear mixtures, ***X***. The key of this method is to find an unmixing matrix, ***W***, which specifies filters that linearly invert the mixing process. Thus, a standard ICA model can be expressed in notation as:

2 $${\boldsymbol{S}}={\bf{W}}{\boldsymbol{X}}$$

To better characterize the spatiotemporal properties, Equation 2 can be adapted as the following:

3 $${\boldsymbol{X}}={{\boldsymbol{W}}}^{-1}{\boldsymbol{S}}$$

Here, ***X*** is an *m* × *n* matrix, of which the *i*th row presents the time courses originally measured (e.g., the time course of one of the facial ROIs in the present study). ***S*** is also an *m* × *n* matrix, of which the *i*th row presents the temporal dynamics of the *i*th independent component (IC). ***W***^−1^ is an *m* × *m* mixing matrix, of which the *i*th column reflects the relative projection strengths of the temporal dynamics of the *i*th independent component. In other words, the *i*th column of ***W***^−1^ indicates the spatial distribution of the *i*th independent component whereby we can find where this independent component can be best reflected.

#### Group temporal independent component analysis

It is well known that there is no ordering of decomposed independent components. Thus, if we perform ICA for each participant separately, it is impossible to make group statistic inferences across all participants. For this reason, we use a group ICA algorithm to solve this problem. This method extends ICA algorithm to the group level and therefore can make group inferences. Due to these advantages, group ICA has been extensively used in processing of various modalities of imaging data such as Electroencephalogram (EEG)^36–38^ and fMRI^39–41^.

As shown in Fig. 2, for each ROI, the time course originally extracted from different participants were first concatenated head-to-tail. Thus, based on Equation 3, the *i*th row of ***X*** presents the concatenated time courses of all participants for the *i*th ROI (Fig. 2b left column). Next, the temporal ICA was performed on these 10 concatenated time courses using Fast ICA algorithm^42^. Then we obtained theoretically 10 independent components, each of which had a concatenated temporal dynamics. Thus the *i*th row of ***S*** indicated temporal dynamics of the *i*th independent component (Fig. 2b right column), whereas the *i*th column of the ***W***^−1^ reflected the spatial distribution of the *i*th independent component that indicated which facial ROIs this independent component was represented on (Fig. 2b middle column). It should be noted that the temporal dynamics of the *i*th IC was also a concatenated pattern of all participants. This concatenated temporal dynamics had the same spatial distribution and therefore was thought to be driven by the same source.

For the concatenated temporal dynamics of each independent component (e.g., the *i*th independent component), it can be segmented into individual temporal dynamics for respective participants. As a result, for the *i*th independent component, different participants had their respective different segmented temporal dynamics but the same spatial distribution. If the temporal dynamics of one independent component contains cardiovascular activity information, then we can reveal where this cardiovascular activity was best represented in a face based on the spatial distribution of this independent component. Thus, using this group temporal ICA method, the time courses originally extracted from ROIs of each participant were decomposed into independent components with fixed orders for all participants.

#### Correlation analysis

After independent components were obtained, to examine which one contained the information of the cardiovascular activity, for each participant, we calculated the Pearson correlation coefficients between the transdermal optical imaging and ECG data in the frequency domain. To do so, first, we transformed both the temporal dynamics of each independent component and the sub-sampled ECG signal (20 Hz) into frequency domain using the Fast Fourier Transformation (FFT). Second, for each magnitude spectrum of each participant, we preserved the frequency components from 0.7 to 2.5 Hz. It is generally accepted that this frequency band contains the cardiac pulse of the majority of the population. Thus, this frequency band selection allows us not only to focus on the main cardiac activity but also to avoid disturbance from other physiological signals such as respiration. Third, for each participant, we calculated the Pearson correlation coefficients between the magnitude spectrum (banded from 0.7 to 2.5 Hz) of temporal dynamics of each independent component and that of sub-sampled ECG signal. Then, we transformed each correlation coefficient to Z-scores using Fisher’s Z-transformation. Finally, for each independent component, one-sample t-tests were performed on the Z scores across the 11 participants to test whether these Z-scores were significantly different from zero at the group level.

Using the same approach as above, we performed the correlation analysis between the phase spectrum of the temporal dynamics of the ICA components and that of the ECG data.

### Data availability

The dataset analyzed during the present study is available from the corresponding author on reasonable request.
